# Economic Costs and Benefits of the Digital OurRelationship Program for Distressed Couples

**DOI:** 10.1111/famp.70177

**Published:** 2026-07-09

**Authors:** Brian D. Doss, Shayna Guttman, Maya Metser‐Waldman, Nirvi Ajmera, Alex Dopp

**Affiliations:** ^1^ Department of Psychology University of Miami Coral Gables Florida USA; ^2^ Rand Santa Monica California USA

**Keywords:** cost offset, cost–benefit, couple intervention, relationship intervention

## Abstract

Relationship distress is a prevalent issue with significant economic and societal consequences, including increased mental and physical health problems, decreased workplace productivity, and increased absences. Although couple therapy has been shown to improve relationship and individual well‐being, its high costs limit accessibility. Digital relationship interventions provide a cost‐effective alternative. However, limited research has assessed the cost–benefit of such programs using experimental designs. This study conducted a cost–benefit analysis of the OurRelationship program using data from two randomized controlled trials (RCTs). The Washington State Institute for Public Policy (WSIPP) cost–benefit model was applied to quantify economic benefits across four domains—depression, anxiety, alcohol misuse, and insomnia—relative to a waitlist control. Program costs were estimated at $280 per individual, and benefits were assessed in terms of healthcare savings, productivity gains, and labor market outcomes. The primary analysis found that, for every dollar spent on the OurRelationship program, $6.60 was saved in societal costs. Sensitivity analyses suggested benefit–cost ratios ranging from $1.88 to $22.06 depending on assumptions regarding the number of participants, duration of effects, and magnitude of program impact. Findings suggest that the OurRelationship program yields substantial economic benefits, making it a viable investment for policymakers, employers, and healthcare systems. The study supports broader implementation of efficacious digital relationship interventions and highlights the importance of economic evaluations in guiding funding decisions for relationship support programs. Further research should explore the long‐term benefits and applicability across diverse populations.

## 1 Introduction

The dissolution rate of romantic relationships in the United States (and in most countries around the world) indicates the need for interventions that can support healthy relationship functioning. Despite intending to spend the rest of their lives together, over half of marriages end in divorce (Centers for Disease Control and Prevention 2015) or are currently distressed (Whisman et al. 2008). Breakup rates of cohabiting couples—to whom as many as 40% of children in the United States are born (Osterman et al. 2026)—are even higher (Bramlett and Mosher 2002). The implications of this relationship health crisis are on par with the mental health and physical health crises in the United States.

Unlike these other health domains, discussion of relationship distress tends to be limited to its impact on personal happiness, social instability, and family functioning. What typically remains hidden is the massive financial impact of relationship distress and divorce. Understanding the societal effects of romantic relationship distress is complex because we cannot place inherent value on any given relationship outcome (e.g., ending vs. continuing the relationship). That said, the optimal relationship outcome from a health perspective is the one that maximizes physical and mental health outcomes for all parties in the relationship—and these outcomes are strongly affected by relationship distress. For example, relationship distress increases subsequent rates of depression, anxiety, substance use disorders, immune functioning, sleep, cardiovascular health, and global perceived health (Robles et al. 2014; Whisman 2007).

Furthermore, previous studies have demonstrated that relationship‐related health functioning produces societal economic outcomes by impacting functioning in areas such as employee functioning, use of physical and mental health services, and need for government assistance. Relationship distress and breakup are negatively associated with employee performance (Forthofer et al. 1996), including an 18% decrease in work performance, a 20% increase in work related exhaustion, and a 26% increase in strained work relationships. Indeed, marital distress results in a loss of one workday a month in increased absenteeism and reduced productivity (Peasley et al. 2020). A divorce experienced by senior leadership can affect the entire company; for example, companies showed a 1.2%–3.1% decrease in the operating return on assets (i.e., income earned on their resources) in the year after their CEO experienced a divorce (Kleindienst et al. 2022). Health system expenditures are also increased by relationship distress and divorce. In a survey of over 30 large Employee Assistance Programs (EAPs) in the United States (US) and Canada, the presenting issue in 12% of counseling appointments was marital problems—more frequent than depression, anxiety, substance misuse, or stress (Attridge 2022). Additionally, health care costs are 46% higher for those who are unhappy in their marriages compared to those who are happily married (Prigerson et al. 2000). Finally, federal and state governments experience economic impacts from relationship distress and divorce. For example, the cost of divorce in Texas on means‐tested government assistance—such as medical, food, housing, and utility assistance—has been estimated at more than $4 billion dollars annually in state and federal funds (Schramm et al. 2013; figure adjusted for inflation to 2024 dollars).

### 1.1 Effects of Relationship Interventions

Fortunately, there are effective relationship interventions that improve relationship functioning. Preventative interventions have been generally shown to have small‐sized effects (Hawkins et al. 2008), brief secondary in‐person and digital interventions for couples having relationship problems have small‐to‐medium‐sized effects (Cigrang et al. 2022; Doss et al. 2016; Doss, Knopp, et al. 2020), and more intensive couple therapy has medium‐to‐large‐sized effects on relationship satisfaction (Doss et al. 2012; Roddy, Walsh, et al. 2020).

Although the effects of relationship interventions on individual functioning have not been explored as frequently, studies have shown that effective relationship‐focused interventions can improve multiple domains of behavioral health. Indeed, couple therapy and brief digital couple interventions significantly reduce depressive symptoms (Barbato and D'Avanzo 2020; Doss et al. 2016) and anxious symptoms (Doss et al. 2016; Lenger et al. 2024). Moreover, a Cochrane systematic review and meta‐analysis (Barbato and D'Avanzo 2020; Barbato et al. 2018) found that couple‐based treatment for depression was equally effective as individual therapy in reducing depression but more effective in ameliorating relationship distress—which in turn reduces depression severity and relapse risk following therapy. Additionally, several studies of brief in‐person couple interventions improved sleep quality (Adler‐Baeder et al. 2022, Barton et al. 2021). Relationship interventions also have the potential to significantly improve substance misuse. Indeed, couple therapy tailored for couples in which one partner was experiencing substance misuse significantly improves substance‐related problems (Powers et al. 2008).

### 1.2 Economic Impact of Relationship Interventions

Understanding the extent that relationship interventions offset the societal economic impacts of relationship distress can help guide policy decisions around investment in these interventions. To date, most research has examined couple therapy tailored to improve individuals' mental health problems. For example, every dollar spent on couple therapy for alcoholism yielded $5.87 in savings in subsequent medical and legal costs (O'Farrell et al. 1996). Broadening the scope to family therapy, every dollar spent on a family treatment for youth with anxiety disorders yielded a cost savings of $1.73 (Slade et al. 2021). Similarly, family therapy for schizophrenia was associated with a $1962 decrease in subsequent general medical and hospitalization costs (Christenson et al. 2014).

Some initial research has even suggested that couple therapy focused on relationships (rather than on individual‐level mental health disorders) can also result in cost offsets. Couple therapy delivered as part of a Health Maintenance Organization significantly reduced subsequent health care utilization (Law and Crane 2000). Similarly, in the Veterans Health Administration, couple therapy significantly reduced subsequent mental health and physical health (and their associated costs) – especially for high utilizers of those services and when couples completed the therapy (Madsen et al. 2017).

However, a full course of couple therapy is expensive. Briefer couple interventions—even if not as effective as more intensive couple therapy—may produce more benefits per dollar spent. For example, although a digital intervention with coach support was approximately 50% less likely to create reliable improvements in relationship satisfaction than was couple therapy, the cost of delivering that digital program was only 4‐8% of the cost of providing 6 months of couple therapy (Georgia Salivar, Rothman, et al. 2020). Additionally, group‐based relationship education over the transition to parenthood has been shown to lead to fewer cesarean sections (Feinberg et al. 2015) and even fewer low‐weight births (Rhoades et al. 2022)—a cost savings of approximately $76,153 from a program costing about $2000 per person.

Unfortunately, these studies have generally relied on examinations of cost offsets (reductions in other costs) or cost‐effectiveness (the relative costs of improvements from two relationship interventions). However, when an organization or insurance plan is considering covering an intervention, the cost–benefit of that intervention (whether the savings resulting from the intervention are greater than the cost of providing the intervention) is essential information.

Additionally, virtually all the studies examining the cost‐effectiveness or cost–benefit of relationship interventions have relied on correlational or within‐group designs to estimate an intervention effect—which makes it difficult to disentangle the effects of the intervention from natural remission and other validity threats. The only between‐group cost–benefit analysis of relationship education and couple therapy we are aware of relied primarily on correlational associations between relationship and individual functioning to calculate a cost–benefit ratio (Shamblen et al. 2018), likely overestimating the economic benefit of the interventions. Therefore, as noted in the Cochrane review of couple interventions for depression, future economic evaluation questions should be addressed using data from experimental designs (Barbato and D'Avanzo 2020; Barbato et al. 2018).

### 1.3 Current Study

The aims of the current study are: (a) to conduct a cost–benefit analysis to determine the cost–benefit ratio of a brief relationship intervention (vs. waitlist control) and (b) to conduct sensitivity analyses to determine how the cost–benefit ratio was affected by uncertainty in the values of key model parameters. We chose to evaluate the OurRelationship program—a low‐intensity, digital intervention—because of previous research showing its superior cost‐effectiveness relative to evidence‐based couple therapy (Georgia Salivar, Rothman, et al. 2020). Additionally, the federal government provides the OurRelationship program through the Healthy Marriage initiative of the Administration for Children and Families.

The OurRelationship program consists of 8–10 h of digital content adapted from in‐person Integrative Behavioral Couple Therapy (IBCT; Christensen et al. 2010). In addition to the online content, couples have four 20‐min calls with a project coach. Couples also receive email and text reminders about activity completion/non‐completion, certain key responses in the program, and upcoming coach calls. Interested readers can refer to previous publications (Doss et al. 2013) for a more detailed description of the OurRelationship program.

The OurRelationship program has been tested in several randomized controlled trials (RCTs), including nationwide samples (Doss et al. 2016), low‐income (Doss, Knopp, et al. 2020; Doss, Roddy, et al. 2020), same‐gender samples (Nowlan et al. 2026), and military samples (Georgia Salivar, Knopp, et al. 2020). Across these RCTs, the program has been shown to have significant effects on multiple domains of relationship functioning relative to a waitlist control group. For example, the program improves relationship satisfaction (*d* = 0.53 to 0.69), communication conflict (*d* = −0.33 to −0.78), and coparenting conflict (*d* = −0.27). In the current study, we focused on effects in four domains of individual functioning—depression, anxiety, alcohol misuse, and insomnia—as they are the outcomes that have been studied in RCTs of the OurRelationship program with the clearest ties to financial benefits in previous cost–benefit research.

In the current study, we modeled the cost–benefit of OurRelationship versus waitlist control using the Washington State Institute for Public Policy (WSIPP) comprehensive cost–benefit analysis model (hereafter, WSIPP model; see Washington State Institute for Public Policy 2016). WSIPP is a nonpartisan public research group that has been recognized nationally and internationally for the depth and quality of its benefit‐costs analyses. Specifically, we applied the WSIPP model to cost and outcome data from two previous RCTs: (a) Study 1: a national study of relationally‐distressed couples (Doss et al. 2016) and (b) Study 2: a national sample of low‐income couples (Doss, Knopp, et al. 2020; Doss, Roddy, et al. 2020; Roddy, Rhoades, and Doss 2020). Other RCTs of the OurRelationship program were omitted because they focused exclusively on relationship outcomes or because they examined outcomes in specific populations of couples (e.g., military couples, couples with young children). Across both studies, we calculated the economic benefits of OurRelationship in reducing expenses to (a) participants (e.g., health care expenditures, lost productivity); (b) taxpayers (e.g., labor market, public health care costs); and (c) society (e.g., links to crime victimization and education outcomes).

## 2 Method

### 2.1 Description of Original Studies

This study utilizes data from two randomized controlled trials (RCTs) of the OurRelationship program. Both studies were approved by the University of Miami Social Sciences Institutional Review Board. The program's effects on depressive and anxious symptoms are taken from Study 1 (Doss et al. 2016), which lasted 1.7 years and involved 300 heterosexual couples (151 in the program and 149 in the control group). The sample was generally representative of the United States in terms of race, ethnicity, and education level. Average relationship length was just under 10 years (M = 9.72 years; SD = 8.34), and 80% of couples were married. Participants were on average 36.11 years old (SD = 9.58), and 73% had one or more children living in the household. To be eligible, at least one member of the couple needed to score in the distressed range on a measure of relationship satisfaction.

Estimates of OurRelationship's effect on insomnia and problematic alcohol use are derived from Study 2 (Roddy, Rhoades, and Doss 2020), a 1.9‐year RCT (*N* = 495 couples). In this study, over 85% of the sample fell at or below 200% of the federal poverty line and were generally representative of low‐income couples in the United States in terms of race and education; however, the study somewhat underrepresented Hispanic couples, perhaps because enrollment was limited to those fluent in English. Most couples were of different genders (93%) and married (52%); they had been together an average of 6.14 years. Data used in the current study are from 248 couples randomized to the OurRelationship program and 247 couples randomized to the waitlist control condition.

### 2.2 Measures

The Center for Epidemiologic Studies–Depression (CES‐D) 10‐item scale was used, as it was developed to assess depressive symptoms in community samples (Cole et al. 2004). All items were self‐reported on a 0–3 Likert scale with higher scores indicating more symptoms. Cronbach's alpha was 0.84. Of a total possible range of 0–30, participants' actual scores ranged from 0 to 30 (Doss et al. 2016).

The Generalized Anxiety Disorder (GAD)‐7 scale was used to assess anxiety symptoms (Spitzer et al. 2006). It has high test–retest reliability (ICC = 0.83) and discriminates well between depression and anxiety symptoms. All items were self‐reported on a 0–3 Likert scale (range 0–21) with higher scores indicating more symptoms. Cronbach's alpha was 0.91 and participants' actual scores ranged from 0 to 21 (Doss et al. 2016).

The 8‐item NIH PROMIS Alcohol Use measure was used to assess problematic alcohol use in the past month (Pilkonis et al. 2013). Participants were first asked if they have used alcohol in the past 30 days (scored 0 if no use). If alcohol use was endorsed, participants then responded to seven additional questions scored on a 1‐ to 5‐point Likert scale, with higher numbers indicative of greater problems (range 0–35). Participants' actual scores ranged from 0 to 35, and alpha was 0.91 (Roddy, Rhoades, and Doss 2020).

The 7‐item Insomnia Severity Index (ISI; Bastien et al. 2001) was used to assess insomnia symptoms. ISI scores have been shown to be highly correlated with objective polysomnography, sleep diaries, as well as clinician and partner ratings (Bastien et al. 2001). Responses were self‐reported on a 0‐ to 4‐point Likert scale (range 0–28), with higher scores indicating greater insomnia symptoms. Participants' actual scores ranged from 0 to 28; alpha was 0.86 (Roddy, Rhoades, and Doss 2020).

### 2.3 Cost–Benefit Analysis Approach

#### 2.3.1 Benefit Calculations

Benefit estimates for depressive symptoms, anxious symptoms, and problematic alcohol use were extracted from WSIPP's (Washington State Institute for Public Policy 2016) benefit–cost model. The 2016 estimates were used because they approximated the median time of intervention delivery across the two samples. For all three outcomes, benefits were calculated across two categories: (a) labor market and VSL (i.e., value of a statistical life) effects and (b) health care costs. Benefit estimates of insomnia outcomes were not included in WSIPP but we were able to monetize those outcomes using comparable methods and integrate the results into cost–benefit estimates generated by the WSIPP model. Specifically, we extracted the predicted (i.e., adjusted) estimates of healthcare and productivity costs for individuals with no insomnia and moderate/severe insomnia (Sarsour et al. 2011) and adjusted for inflation.

We assumed that the effect sizes decay over an individual's lifetime, with decay rates derived from existing literature on symptom trajectories over time. For example, the WSIPP model assumes that 48% of benefits related to depression and anxiety are lost every 2 years, whereas 86% of benefits related to problematic alcohol abuse are lost every 2 years.

#### 2.3.2 Cost Calculations

Costs of providing OurRelationship were calculated across six domains: ongoing website expenditures, coaching, supervision, customer support, rent/facilities, and equipment. There were two types of costs that were not included in these calculations, as we consider in more detail in the Discussion. First, the cost for initial development of the program was not included in cost estimates because these expenses are not incurred by outside organizations during program delivery. Second, marketing costs were not included because OurRelationship will likely be offered as part of a suite of wellness services (and thus share advertising expenses with other programming).

All costs were totaled separately for each RCT and converted into per‐individual costs, after which we calculated an overall average per‐individual. Costs were then adjusted to 2024 dollars using a Consumer Price Index inflation calculator (U.S. Bureau of Labor Statistics 2025). There were no costs involved in the control condition so the total cost of OurRelationship was the same as its incremental cost over the waitlist control. Except for rent, all cost figures were obtained from actual grant expenditures during the two studies. Rent was not charged directly because the studies were run from a university. Instead, rent costs were estimated using office space rent prices from the Commercial Industrial Association of South Florida for Coral Gables, FL (Commercial Industrial Association of South Florida 2013, 2016). Costs were calculated based on the square footage of the office from which the study was conducted. More specifics on cost calculations are available in the Supporting Information.

#### 2.3.3 Cost–Benefit Calculations

We summed all included benefits across outcomes to calculate the total estimated benefits per individual. We then subtracted the incremental cost per individual from the benefit estimate to obtain the net benefit of OurRelationship over the waitlist control condition; a positive net benefit indicates benefits that exceeded the costs. We also divided the total estimated benefits by the cost per individual to obtain the benefit–cost ratio for OurRelationship; this ratio indicates the amount of benefits expected for each dollar spent on OurRelationship, with values greater than 1.00 also indicating benefits that exceeded the costs.

Finally, in addition to point estimates of the cost–benefit ratio, it is important to conduct sensitivity analyses to determine the impact that uncertainty (i.e., plausible range of variation) in key model parameters have on that estimate. In the present study, we examined four key parameters through sensitivity analysis: (a) the magnitude of the intervention's effect size, (b) the approach to reconciling benefits across outcomes to avoid double‐counting effects, (c) the duration of the benefits, and (d) the number of couples served.

### 2.4 Sensitivity Analyses

#### 2.4.1 Magnitude of Effects

##### 2.4.1.1 Primary Estimate

Effects of the Our Relationship program on these four domains were determined by taking the between‐group effect size Cohen's *d* from the original RCTs. Specifically, the effect sizes were: (a) depression: −0.50 (SE = 0.083), (b) anxiety: −0.21 (SE = 0.082), (c) problematic alcohol use: −0.11 (SE = 0.063), and (d) insomnia: −0.24 (SE = 0.064).

##### 2.4.1.2 Sensitivity Analyses

Sensitivity of the cost‐saving estimates to variation in the effect of the program was calculated by utilizing the upper and lower 95% confidence interval for the effect size (*d*) estimates for depression (−0.34 to −0.66), anxiety (−0.05 to −0.37), alcohol misuse (−0.01 to −0.23), and insomnia (−0.11 to −0.37).

#### 2.4.2 Overlap of Benefits Across Outcomes

To prevent double counting the effects of interrelated program outcomes on the same benefits category (e.g., labor market, health care), we calculated the percent of variance in change in each primary outcome (depression, anxiety, alcohol use, insomnia) not accounted for by change in outcomes already entered in the model. We then adjusted (penalized) the cost savings of each outcome by multiplying the cost savings estimate by the estimate of its independent variance after accounting for all variables already in the model. For example, we only included the cost benefit of problematic alcohol that could not be accounted for by depressive and anxious symptoms (the two variables already in the model).

When calculating the shared variance estimates, the order of entry for calculations was based on the order in which the WSIPP model (Washington State Institute for Public Policy 2016) would have evaluated those outcomes for overlap, starting with the largest estimated benefit (see Sensitivity Analyses for details of how the model handles this); we always entered insomnia benefits last because they were not part of the original WSIPP model, and therefore we could not determine where they would fall in the order. For benefits from labor market savings and VSL, anxiety had the largest estimated benefits and so was entered into the model first (followed by depression, then problematic alcohol use, then insomnia). However, for benefits from health care savings, depression had the largest estimated benefits, so it was entered first (followed by anxiety, problematic alcohol use, and insomnia).

##### 2.4.2.1 Primary Estimates

Changes in anxious and depressive symptoms showed substantial overlap of 69% (*R* ^2^ = 0.694) but changes in problematic alcohol use were more independent of changes in anxious and depressive symptoms (combined *R* ^2^ = 0.017). Overlap of insomnia symptoms with the other three changes was moderate (*R* ^2^ = 0.227).

##### 2.4.2.2 Sensitivity Analyses

As an estimate of the lower bound of reconciled benefits, we used the WSIPP “trumping” procedure that only includes the highest benefit estimate for each category of benefit (omitting the benefit estimates for the other outcomes entirely). As an estimate of the upper bound of reconciled benefits, we assumed that all benefits were independent (i.e., there was no overlap between outcomes).

#### 2.4.3 Duration of Effects

##### 2.4.3.1 Primary Estimates

Primary estimates of duration of effects were calculated assuming that decay of those benefits (as previously described) begins immediately following the program.

##### 2.4.3.2 Sensitivity Analyses

The estimate of the lower bound of the duration was the same as the primary estimate (decay starting at the end of the program) because analyses of the OurRelationship program have consistently found that couples continue to improve from the middle to the end of the program (Doss et al. 2016; Doss, Knopp, et al. 2020; Doss, Roddy, et al. 2020). The upper‐bound estimate used the (generally minimal) within‐group changes observed in the OurRelationship group through 12 months after the end of the program (Doss et al. 2019; Roddy et al. 2021) to estimate decay of benefits during the first year following the program (Between‐group estimates were not available for the follow‐up period because couples in the waitlist control group received OurRelationship after the 2‐month intervention period). After 12 months following the end of the program, the standard WSIPP decay rates were applied.

#### 2.4.4 Number of Couples

The range of couples served was calculated using the same or similar trials of the OurRelationship program delivered under a similar cost structure (e.g., with the same level of staff support and hosting/maintenance by the same company). These values were used to calculate the per‐couple cost of OurRelationship based on the number of couples served in a 12‐month period.

##### 2.4.4.1 Primary Estimates

For our main analyses, we utilized the mean number of couples served per year in the studies from which the effect sizes were obtained (*n* = 110 couples per year receiving OurRelationship).

##### 2.4.4.2 Sensitivity Analyses

The lower‐bound of couples served was derived from the fewest number of couples ever served per year in a trial of the OurRelationship program (*n* = 89 couples per year). The upper‐bound of couples served was conservatively calculated as one third of the couples served in the most recent year (2024) of OurRelationship services in a nationwide project (*n* = one third of 1146 couples, or 382 couples). One third of couples served was used rather than the full number of couples served because costs required to serve the full number are higher (e.g., more coaching time, a second full‐time coordinator).

## 3 Results

### 3.1 Main Analyses

#### 3.1.1 Costs

As shown in Table 1, the average cost per participant in 2024 dollars was $326 for Study 1 (*n* = 302, or 151 couples) and $252 for Study 2 (*n* = 496, or 248 couples). Adjusted for inflation, the weighted average cost per individual across the two studies was $280.

**TABLE 1:** Costs of providing OurRelationship in each randomized trial.

Category	Study 1 (Doss et al. 2016)	Study 2 (Roddy, Rhoades, and Doss 2020)
Website costs		
Maintenance, licensing, hosting of LMS	$10,800	$ 15,600
Domain name	$ 32	$ 34
Hosting of public site	$ 227	$ 244
Coach costs		
Initial training	$ 972	$ 2340
Weekly group supervision	$10,660	$ 12,636
Coaching sessions	$ 9173	$ 14,508
Supervisor costs		
Weekly group supervision	$16,180	$ 17,784
Grad student peer supervisor (4 h/week)	*N/A*	$ 6469
Customer support		
Participant coordinator (salary & benefits)	$38,514	$ 39,348
Rent		
Office space	$ 9635	$ 14,522
Equipment		
Computer + monitor	$ 2132	$ 1719
Total	$98,325	$125,204
Number of Individuals	302	496
Cost Per Individual	$326	$252
Weighted Cost Per Individual	$280	

*Note:* All costs are in 2024 dollars. A full description and calculation of costs can be found in the Supporting Information.

#### 3.1.2 Benefits

Benefit estimates were obtained from Washington State Institute for Public Policy (2016) benefit–cost model. In 2024 dollars, benefits attributable to decreases in anxiety ($790 per individual) were largest followed by insomnia ($386 per individual), alcohol misuse ($366 per individual), and depression ($304 per individual). The total benefits estimated were $1847. Most (63%) of the benefits were to participants or employers, 21% were to taxpayers, and 16% were to the broader society. Full benefit estimates can be found in Table S1.

#### 3.1.3 Cost–Benefit Analysis

The primary analysis indicated that the benefit–cost ratio for the OurRelationship program was estimated to be $6.60 for every $1.00 spent—or a net present value of $1847 per individual in 2024 dollars. Detailed information about the benefit–cost ratios and net present values calculated under various assumptions with the WSIPP model is presented in Table S2.

### 3.2 Sensitivity Analyses

**FIGURE 1:**
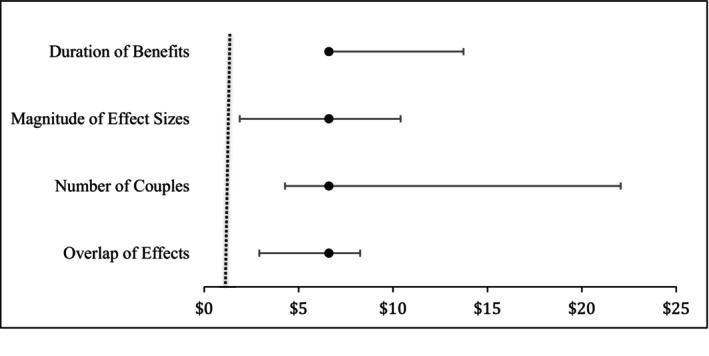
Sensitivity Analysis of Benefit–Cost Ratio. The dots represent the cost–benefit ratio from the primary analysis and the error bars represent the credible ranges of the cost–benefit ratio for the various assumptions made in separate sensitivity analyses. The vertical dotted line represents the cost–benefit ratio of 1, which represents an even return on investment (i.e., the cost and the benefit of providing the intervention are the same).

As depicted in Figure 1, delivery of the OurRelationship program resulted in benefits that exceeded the program costs (i.e., positive net present value and benefit–cost ratio over 1.00) in all cases. At a minimum, with the lowest plausible value for effect sizes, the program was estimated to produce $1.88 in benefits for every $1 spent delivering the program. At the maximum, with the highest plausible value for the number of couples served in a year, the program was estimated to produce $22.06 in cost savings for every $1 spent. Detailed information about the benefit–cost ratios and net present values calculated in the sensitivity analyses are presented in Table S2.

## 4 Discussion

The present study suggests that every dollar spent on delivering the OurRelationship program yields savings of approximately $6.60 in increased healthcare expenditure, increased absenteeism, and reduced productivity. This cost savings is consistent with previous estimates of cost savings in subsequent medical and legal costs for couple therapy for alcoholism ($5.87; O'Farrell et al. 1996) and with the cost offsets of group‐based couple and family interventions (Feinberg et al. 2015; Rhoades et al. 2022).

In interpreting this overall cost–benefit ratio, it is important to consider the conservative assumptions made in the benefit estimates that comprise it. We omitted from the cost savings estimates demonstrated effects of the program on co‐parenting, child internalizing symptoms, and child externalizing symptoms (Doss, Roddy, et al. 2020) and global perceived health (Doss et al. 2016). Additionally, we omitted cost savings of future relationship deterioration to the government. Previous reports (Schramm et al. 2013) have estimated that, in the state of Texas alone, divorce costs taxpayers over $4 billion dollars a year from increased social services for couples who fall under the poverty level following a divorce.

To examine the robustness of the original cost–benefit ratio to various assumptions, we conducted a series of sensitivity analyses. Across all sensitivity analyses, providing the OurRelationship program yielded a cost savings; estimates ranged from a cost savings of $1.88 to $22.06 for every dollar spent on the OurRelationship program. As with the primary estimates, these analyses used conservative estimates of costs and benefits. For example, the upper bound of program effects were calculated from estimates showing that changes in the program are relatively stable for at least a year after the program (and decayed after 12 months). However, it is possible that program effects are stable for substantially longer. For example, effects for the couple therapy approach from which the OurRelationship program was adopted are relatively stable for as much as 5 years after the end of therapy (Christensen et al. 2010). Similarly, the lower bound of the effect size yielded the lowest cost‐savings of all sensitivity analyses. However, the OurRelationship program's effects have been largely replicated in several RCTs across various types of couples (e.g., Georgia Salivar, Knopp, et al. 2020; Hatch et al. 2021; Mitchell et al. 2023). As such, using the lower bounds of the 95% confidence interval to calculate minimum effects is likely conservative.

However, there are two cost categories that organizations may need to consider that were not included in our estimates. First, we assumed that organizations, businesses, and governments would not be interested in building their own digital programs and would instead adopt existing, empirically‐supported interventions. Therefore, we did not include program development costs. Second, we did not include marketing costs in our estimates. In many wellness, benefit, or EAPs, a relationship offering would likely be integrated into an existing suite of services and not substantially increase marketing costs above and beyond the marketing that was already been done. Similarly, some governments might rely primarily on existing referral networks (e.g., 211 phone numbers; online referral platforms). However, some organizations may incur additional advertising expenses; in this case, their cost savings would be reduced.

### 4.1 Limitations and Future Directions

Despite the robustness of the cost–benefit estimates, there are several limitations which should be considered. Most importantly, estimates of the benefits were extracted from the WSIPP database rather than collected from the couples who participated in the OurRelationship program. It is possible that the links between the program outcomes and the benefits are different in distressed couples than they are in the studies utilized by the WSIPP model. Further, the rates of decay of benefits from couple interventions may be different than the rates of decay of benefits from individual interventions utilized in the WSIPP model.

Our reliance on self‐report measures to assess program benefits is also a limitation. For example, although our measure of insomnia is significantly correlated with polysomnography, sleep diaries, and partner reports of sleep (Bastien et al. 2001), utilization of an alternative methodology may have helped minimize the overlap with other self‐reported measures. Additionally, utilizing structured clinical interviews, rather than self‐reports of symptom counts, would have strengthened our confidence in the program's effects on depressive and anxious symptoms.

Additionally, the clinical trials from which benefits and cost estimates were obtained were relatively small (Total *N* = 399 couples). Both estimates should be replicated in future studies of the OurRelationship program before firm conclusions are drawn. It would also be informative to replicate these findings with in‐person or synchronous online brief interventions.

Finally, these studies were not explicitly designed for a cost–benefit analysis. Instead, costs were obtained from records of grant expenses and publicly‐available databases (e.g., for rent costs). Additionally, it was not always possible to definitively separate costs attributable to delivery of the program from those attributable to the research study (which we sought to exclude from cost estimates).

### 4.2 Implications

These findings have important implications for multiple stakeholders interested in making investments in programming to support healthy relationships and reduce relationship distress. For governments and policymakers, the results show that supporting effective and affordable relationship education can have societal‐level benefits and cost‐savings. Relationship conflict and divorce convey enormous costs resulting from need‐based services such as food, medical, housing, utility, and cash assistance, with one study estimating it consumes more than 10% of state budgets (Schramm et al. 2013). Fortunately, at the federal level, the Administration for Children and Families (Healthy Marriage and Responsible Fatherhood 2025) provides relationship support. At the state level, states like Texas (Twogether in Texas 2025) and Utah (Utah State University Extension 2025) encourage participation in relationship education by providing a discount on marriage license fees. However, more could be done to implement and support a stepped‐care model, where services like broad relationship education, OurRelationship, and couple therapy are funded to help couples experiencing relationship problems.

For employers, EAPs, and insurance plans, additional research would be useful to understand the budget impact for specific payers of investing in relationship services. Given that over 80% of the estimated benefits in the current study arise from improvements in productivity and reductions in absenteeism, results suggest that there would be direct cost savings to employers by providing relationship services. For EAPs and insurance plans, the primary cost savings may result from decreased utilization of more expensive couple therapy. Indeed, approximately half of couples who complete the OurRelationship program no longer need couple therapy (Trillingsgaard et al. 2023).

We are aware that these organizations' budgets and their ability to manage various offerings are often constrained. However, providing relationship programs to support distressed romantic relationships has broad preventive and ameliorative effects—improving not only relationships but individuals' productivity (Doss et al. 2016), behavioral health (Roddy, Rhoades, and Doss 2020), parenting, and child functioning (Doss, Roddy, et al. 2020). In recent years, organizations have begun to expand coverage to families and children for assistance for new parents and caregiving. The results of the present study suggest that support for distressed relationships and divorce would be an important addition given that approximately half of employees will experience a divorce or breakup of a long‐term relationship (Centers for Disease Control and Prevention 2015) at some point during their career and an additional 31% percent are likely to experience substantial relationship distress (Whisman et al. 2008).

Finally, these findings have implications for research on couple interventions. For organizations like the National Institutes of Health, these findings highlight the benefits of interventions that target a broader system (not just an individual). For researchers, these findings are a first step in quantifying the cost benefits of improvements in distressed couples' relationship functioning. Because the OurRelationship program does not include an explicit focus on any of the individual outcomes included in this study, researchers may be able to utilize these findings to estimate how improvements in various components of relationship functioning (e.g., a weighted average of Cohen's *d* = 0.59 improvement in relationship satisfaction) can be converted into a cost offset (e.g., $1820 per individual in reduced health care costs and gains in productivity and labor market effects). While these estimates will admittedly be rough approximations, they may help couple researchers conceptualize the value of investing in relationship functioning. Furthermore, we hope that researchers will conduct similar cost–benefit studies on other relationship interventions. These studies would ideally expand the number of intervention outcomes that are monetized and collect benefit information in the same sample.

## Funding

The studies from which the effect and cost data were derived were funded by previous federal grants from the US government: U.S. Department of Health and Human Services, Administration for Children and Families, Grant 90FM0063 and National Institutes of Child Health and Human Development (R01HD059802). Any opinions, findings and conclusions or recommendations expressed in this material are those of the authors and do not necessarily reflect the views of the U.S. Department of Health and Human Services, Administration for Children and Families or the National Institutes of Health.

## Conflicts of Interest

Dr. Doss is a coinventor of the OurRelationship program and an equity owner in OurRelationship LLC. His conflicts of interest is managed by a COI plan overseen by the University of Miami. Dr. Dopp was involved in the study as a paid external consultant to help further manage this conflict of interest. Dr. Dopp conducted all of the benefit and cost–benefit analyses and wrote or approved the corresponding sections of the manuscript. The other authors are graduate students at the University of Miami and have no employment or financial relationship with OurRelationship LLC.

## Supporting information


**Table S1:** Benefits by outcome and source.
**Table S2:** Cumulative benefits of OurRelationship Program.

## Data Availability

The data that support the findings of this study are available from the corresponding author upon reasonable request.
