# Supplementary material for: Economic Costs and Benefits of the Digital OurRelationship Program for Distressed Couples
Source: Fam Process. 2026 Jul 9;65(3):e70177. doi: 10.1111/famp.70177 (PMC13347763; doi:10.1111/famp.70177)
Supplement: Supplementary file 1 — Table S1: Benefits by outcome and source. Table S2: Cumulative benefits of OurRelationship Program. [file FAMP-65-0-s001.docx]

**Supplemental Materials**

**Additional Information on Cost Calculations**

Ongoing website expenditures consisted of hosting, maintenance, and licensing contracts for the underlying learner management system (Study 1: $10,800; Study 2: $15,600), domain name registration (Study 1: $19 per year * 1.7 years = $32; Study 2: $18 per year * 1.9 years = $34), and public website hosting (Study 1: $134 per year * 1.7 years = $227; Study 2: $129 per year * 1.9 years = $244).

Coaching costs included initial training (Study 1: 4 coaches * 6 hours * $41 hour = $972; Study 2: 6 coaches * 10 hours * $30 hour = $2,340) and weekly group supervision (Study 1: 66 meetings * 4 coaches * $41 an hour = $10,660; Study 2: 54 meetings * 6 coaches * $39 an hour = $12,636). Coaching costs for delivering the intervention to couples (including reschedules, no-shows, and time for email responses) were also included (Study 1: 151 couples * 1.5 hours * $41 an hour = $9,173; Study 2: 248 couples * 1.5 hours * $39 an hour = $14,508).

Costs for the doctoral-level clinical supervisor to provide weekly group supervision and occasional individual consultation were included for both studies (Study 1: $135 per hour * 1.5 hours/week * 66 weeks = $16,180; Study 2: $195 per hour * 1.5 hours/week * 54 weeks = $17,784). Additionally, in Study 2, we utilized a senior graduate student as a peer supervisor to provide additional one-on-one supervision. Costs for the peer supervisor were calculated as 20% of the annual stipend of $30,389 and 20% of the annual insurance fee of $1,960 (total = $6,469).

During both studies, a full-time participant coordinator was employed to randomize couples to condition, respond to couples’ and coaches’ emails / phone calls, send reminders for research surveys, and process payments for research survey completion. We calculated that approximately 75% of the coordinator’s responsibilities were attributable to program services (and 25% limited to the underlying research study, which were omitted from these calculations). Salary was included for both studies (Study 1: 75% of $40,119 salary and $11,233 fringe for a total of $38,514; Study 2: 75% of $40,989 and $11,476 fringe, for a total of $39,348).

Rent was not charged directly because the studies were run from a university. Instead, rent costs were estimated using office space rent prices from the Commercial Industrial Association of South Florida for Coral Gables, FL [61-62]. Costs were calculated based on the square footage of the office from which the study was conducted (Study 1: $46.45 per square foot * 122 square feet * 1.7 years = $9,635; Study 2: $49.63 per square foot * 154 square feet * 1.9 years = $14,522).

Finally, a computer and monitor were purchased for the participant coordinator during Study 1 ($2,132) and Study 2 ($1,720).

**Table S1**

*Benefits by Outcome and Source*

| **Outcome** | | **Participant** | **Taxpayer** | **Societal** | **Total** |
| --- | --- | --- | --- | --- | --- |
| **Anxiety** | |  |  |  |  |
|  | Labor Market and VSL | $532 | $158 | $ 84 | **$773** |
|  | Health Care Costs | $ 2 | $ 5 | $ 11 | **$ 17** |
| **Depression** | |  |  |  |  |
|  | Labor Market and VSL | $133 | $ 39 | $ 22 | **$195** |
|  | Health Care Costs | $ 12 | $ 32 | $ 65 | **$110** |
| **Alcohol Misuse** | |  |  |  |  |
|  | Labor Market and VSL | $236 | $ 70 | $ 40 | **$346** |
|  | Health Care Costs | $ 1 | $ 6 | $ 12 | **$ 18** |
| **Insomnia** | |  |  |  |  |
|  | Labor Market and VSL | *N/A* | *N/A* | *N/A* | **$217** |
|  | Health Care Costs | *N/A* | *N/A* | *N/A* | **$169** |

*Note*. VSL = Value of a Statistical Life. Breakdown of insomnia costs by source was not available in the published source (Bastien et al., 2001). Participant costs = Benefits to program participants, such as avoided out-of-pocket health care costs and improved quality of life. Taxpayer costs = Avoided expenses to federal, state, and local taxpayers resulting of program outcomes; for example, decreased use of publicly funded services and health care. Societal costs = Economic effects of the program outcomes beyond participants and taxpayers, including tangible and intangible benefits to crime victims for avoided crime, "spillover" benefits of work productivity to coworkers and employers, and decreased mortality (value of a statistical life).

**Table S2**

*Cumulative Benefits of OurRelationship Program*

| **Analysis** | | **Value** | **Net Present Value^a^**  **(per individual)** | **Benefit-Cost Ratio^b^** |
| --- | --- | --- | --- | --- |
| **Primary Analysis** | |  |  |  |
|  | Primary | Primary | $1,847 | $ 6.61 |
| **Sensitivity Analyses** | |  |  |  |
|  | Duration of Benefits | Minimum | $1,847 | $ 6.61 |
|  |  | Maximum | $3,837 | $13.73 |
|  | Magnitude of Effect Sizes | Minimum | $ 525 | $ 1.88 |
|  |  | Maximum | $2,906 | $10.40 |
|  | Number of Couples | Minimum | $1,196 | $ 4.28 |
|  |  | Maximum | $9,246 | $33.09 |
|  | Overlap of Effects | Minimum | $ 816 | $ 2.92 |
|  |  | Maximum | $2,308 | $ 8.26 |

*Note.* All dollar amounts are in 2024 dollars.

^a^ The difference between the incremental costs and benefits of providing OurRelationship over waitlist control condition.

^b^ The incremental benefit divided by the incremental cost of providing OurRelationship over waitlist control condition.
